# Identifying incident cancer cases in routinely collected hospital data: a retrospective validation study

**DOI:** 10.1186/s13104-019-4726-x

**Published:** 2019-10-21

**Authors:** David E. Goldsbury, Marianne F. Weber, Karen Canfell, Dianne L. O’Connell

**Affiliations:** 1 Cancer Research Division, Cancer Council NSW, PO Box 572, Kings Cross, Sydney, NSW 1340 Australia; 2 Sydney School of Public Health, University of Sydney, Sydney, NSW Australia; 3 Prince of Wales Clinical School, UNSW Medicine, Sydney, NSW Australia; 4 School of Medicine and Public Health, University of Newcastle, Newcastle, NSW Australia

**Keywords:** Cancer incidence, Case ascertainment, Record linkage, Validation, Routinely collected data

## Abstract

**Objective:**

Population-level cancer incidence data are critical for epidemiological cancer research, however provision of cancer registry data can be delayed. We previously reported that in a large population-based Australian cohort, registry-based incidence data were well matched by routinely collected hospital diagnosis data (sensitivities and positive predictive values (PPVs) > 80%) for six of the 12 most common cancer types: breast, colorectum, kidney, lung, pancreas and uterus. The available hospital data covered more recent time periods. We have since obtained more recent cancer registry data, allowing us to further test the validity of hospital diagnosis records in identifying incident cases.

**Results:**

The more recent hospital diagnosis data were valid for identifying incident cases for the six cancer types, with sensitivities 81–94% and PPVs 86–96%. However, 2–10% of cases were identified > 3 months after the registry’s diagnosis date and detailed clinical cancer information was unavailable. The level of identification was generally higher for cases aged < 80 years, those with known disease stage and cases living in higher socioeconomic areas. The inclusion of death records increased sensitivity for some cancer types, but requires caution due to potential false-positive cases. This study validates the use of hospital diagnosis records for identifying incident cancer cases.

## Introduction

Data from population-based cancer registries are generally considered the ‘gold standard’ for identifying incident cases of cancer. However, the provision of cancer registration data can be delayed, and other routine data collections may be available earlier for large-scale research studies. We previously investigated alternative sources for identifying more recent cancer diagnoses in New South Wales (NSW), Australia, using routinely collected, population-based administrative health data [1]. Six of the 12 most common cancers (breast, colorectum, kidney, lung, pancreas and uterus) were well ascertained using routinely collected hospital diagnosis data, with sensitivities and positive predictive values (PPVs) > 80%. We recently obtained cancer registry data for three subsequent years, and these were used to further test the validity of hospital records for identifying incident cancer cases.

## Main text

### Methods

The source population was the Sax Institute’s 45 and Up Study conducted in NSW, Australia. The study methods have been described in detail previously [2]. Briefly, potential participants were sampled from the Medicare enrolment database held by the Department of Human Services (formerly Medicare Australia), which provides near-complete population coverage. People aged 80+ years and those living in rural areas were oversampled. 267,153 participants completed a baseline questionnaire during 2006–2009 and provided consent for researchers to access their health-related records from routinely collected datasets. We analysed data for 266,661 participants, excluding people who withdrew from the study, pilot study participants, those aged < 45 and participants with probable false-positive linkages.

We obtained participants’ diagnosis information for all admitted hospital episodes in NSW from the Admitted Patient Data Collection (APDC), statutory cancer registration data from the NSW Cancer Registry (NSWCR) and cause of death data from the Australian Cause of Death Unit Record File (COD-URF). We analysed APDC data for the period 1 July 2001 to 30 June 2016, NSWCR records for 1994–2013 and COD-URF records for 2006–2015. The records were probabilistically linked by the Centre for Health Record Linkage using a best-practice approach to linkage while preserving privacy [3].

Of interest were people identified in the APDC as incident cancer cases during 2011–2013, when NSWCR data were only available to 2010. We identified potential cases from the diagnoses recorded for each hospitalisation, using the following codes from the International Classification of Diseases 10th Edition (ICD10): breast C50, colorectum C18–C20, kidney C64, lung C34, pancreas C25, and uterus C54–C55. We identified their first record of each cancer type in the APDC and took the admission date of that hospitalisation as the diagnosis date. If the person had a record of the same cancer type in the NSWCR up to 31 December 2010 then they were not considered an APDC-identified incident cancer case, as they would have been identified previously using the existing NSWCR data. All remaining cases in the APDC first identified during 2011–2013 were classified as APDC-identified incident cancer cases.

#### Statistical analysis

For each cancer type, the APDC-identified cases were compared with the NSWCR cases diagnosed during 2011–2013 (for which data are now available), using NSWCR data as the reference ‘gold standard’. A true-positive was defined as an APDC-identified case who was also recorded as a cancer in the NSWCR diagnosed during 2011–2013. Sensitivity was calculated as the proportion of all cases in the NSWCR who were true-positives in the APDC. Specificity was calculated as the proportion of all people who were not identified as cases in the NSWCR and who were also not identified as cases in the APDC. PPV was calculated as the proportion of all APDC-identified cases who were true-positives. We assessed these measures of validity by cancer type, stratifying by age group, sex (where relevant), accessibility/remoteness of residence (distance to service centres) and socioeconomic quintile of place of residence [4], and year of diagnosis, together with sensitivity by spread of disease at diagnosis.

Further, we assessed these measures based on true-positives being within ± 3 months, ± 6 months and ± 12 months of the NSWCR diagnosis date. We estimated sensitivity when NSWCR records for 2011–2013 and all available APDC data (to 30 June 2016) were included. For colorectal cancer, we tested the inclusion of cases with ICD10 code C26 (“Other and ill-defined digestive organs”), as has been described previously [5]. We also assessed endometrial cancers (C54.1) as a separate cancer site instead of being included with all uterine cancers (C54–C55).

Our previous detailed analysis of colorectal and lung cancers showed that no other combination of routinely collected data sources (e.g. death records, government-subsidised medicines from the Pharmaceutical Benefits Scheme) had higher sensitivity and PPV than hospital records [1]. We previously found that when combined with hospital data, inclusion of death records increased the sensitivity with which lung cancer was identified by 3%, but decreased the PPV by 2%. In this analysis we assessed the inclusion of death records as an additional source for identifying cancer cases where the cancer type was the underlying or other/contributing cause of death. Analyses were carried out using SAS v9.4 (SAS Institute Inc.).

### Results

There were similar numbers of cancer cases identified in the cancer registry and hospital data in 2011–2013 (Table 1). For all cancer types the PPVs for the hospital data were > 85%, and were at least as high as that estimated in the 2001–2010 study [1]. The sensitivities were also at least as high as those reported previously, apart from kidney cancer (86% versus 91% previously) and uterine cancer (89% versus 92%), although both were still relatively high and for uterine cancer the 95% confidence interval included the previously reported estimate. For kidney cancer there were 35 cancer registry cases not identified in the APDC: 16 were resident in areas close to state borders so were potentially treated interstate (these hospital data were not available), and for nine others a death certificate was the first notification of a cancer diagnosis.

**Table 1:** New cancer cases identified in 2011–2013 in hospital data, relative to cancer registry data

Cancer type	Cases in NSWCR	Cases in APDC	PPV (95% CI)	Sensitivity (95% CI)
Breast	1270	1305	95% (94–96%)	88% (86–90%)
Colorectal	1334	1383	93% (91–94%)	94% (93–95%)
Kidney	250	259	86% (82–90%)	86% (82–90%)
Lung	842	853	88% (86–90%)	81% (78–84%)
Pancreatic	291	296	90% (86–93%)	86% (82–90%)
Uterine	206	196	96% (93–99%)	89% (85–94%)

Specificity > 99.95% for all cancer types

*APDC* Admitted Patient Data Collection, *CI* confidence interval, *NSWCR* New South Wales Cancer Registry, *PPV* positive predictive value

Further analysis by key factors found little variation by sex (where applicable) or diagnosis year. There were differences by age and spread of disease at diagnosis, and socioeconomic level and geographical remoteness of place of residence. For breast cancer, sensitivity appeared lower for people aged 80+ years (75% versus ~ 90% for all other ages), and for pancreatic and uterine cancers sensitivity was ~ 10% points lower for those aged 80+ than that for all other age groups. PPV varied less by age, apart from that for kidney cancer, which declined with increasing age from 94% for people aged < 60 years to 78% for people aged 80+. For people living in the least socioeconomically disadvantaged areas, sensitivity was higher for pancreatic cancer (by ~ 10%), and to a lesser extent for lung, kidney and breast cancers (by ~ 5%), but there was little difference in PPV. Sensitivity appeared to decline with increasing remoteness of residence for breast cancer (91% for major cities, 87% for inner regional areas, 81% for outer regional/remote), while sensitivity and PPV for kidney cancer were higher by ~ 5% for people in major cities compared with other areas. Sensitivity was generally lower for cases with unknown spread of disease recorded in the cancer registry, with little variation among cases with localised, regional or metastatic disease. The biggest differences were for breast cancer cases with unknown stage, with sensitivity of 35% versus ~ 90% for other breast cancer cases, and 50% versus ~ 85% respectively for lung cancer cases.

The accuracy of diagnosis dates determined from hospital admission data varied by cancer type. The proportion of cases (sensitivity) who were identified in the hospital data within 3 months of the cancer registry diagnosis date ranged from 70% for lung cancer to 92% for colorectal cancer. The respective sensitivities were 75% and 93% within 6 months, and 81% and 94% within 12 months. Using all available APDC data (to June 2016), the sensitivity of the hospital records increased by 7% for lung cancer (to 88%), and by 3% for breast and pancreatic cancers.

For colorectal cancer, we tested the inclusion of ICD10 code C26 for all combinations of hospital records, death records and cancer registry records. This made little difference to sensitivity (at most ± 1%), while PPV decreased by 1% for hospital records and by 3% when death records were included. For endometrial cancers (C54.1), there was much lower sensitivity (77%) and slightly lower PPV (93%) than for all uterine cancers combined (C54–C55). Of the 43 cancer registry cases without a hospital record of endometrial cancer, 16 had a hospital record of C55 “Uterus, part unspecified”.

When death records for 2011–2013 were combined with hospital records, the underlying cause of death data increased the sensitivity by 8% for pancreatic cancer, 5% for lung cancer, 4% for kidney cancer, and 0–2% for the other cancer types, while all PPVs decreased by 1–2%. Also including information on other/contributing causes of death made no difference to sensitivity compared with using the underlying cause of death, however the PPV declined by as much as 5% for kidney cancer (reducing it to 80%). The “false-positive” cases identified from the non-registry data sources often had a cancer registry record for a similar cancer group, such as death from kidney cancer (C64) versus renal pelvic cancer (C65) recorded in the NSWCR.

### Discussion

Overall, we found that hospital diagnosis data were valid for identifying incident cancer cases for these six cancer types. We had previously examined the validity of using administrative health data to identify cancer cases using an earlier time period [1]—this current analysis of more recent data primarily showed slightly higher sensitivity and PPV compared with the earlier study, and only a very few instances of lower sensitivity or PPV.

Sensitivity appeared somewhat higher for cases aged < 80 years, those with known disease stage and for cases living in higher socioeconomic areas. This suggests disparities in access to health services for some population groups with differing levels of hospital utilisation, and so cancer cases identified from hospital data may be slightly biased towards the more advantaged groups. There was evidence that the addition of death records may be useful for some cancer types, but this should be approached cautiously due to the potential increase in false-positive cases.

This study validates the use of hospital diagnosis records to identify incident cancer cases in this cohort. Further, lung cancer cases ascertained by this method in the cohort were used to validate a lung cancer risk prediction tool that combines factors such as age, smoking intensity, body mass index and family history and found it had excellent predictive performance [6]. The use of hospital records will help provide cancer incidence data that are as current as possible, allowing for more timely analyses and greater numbers of cases to increase the power to detect associations.

However, the ideal future scenario would be more timely availability of cancer registry data. At the time of writing the most recent data that could be requested were almost four years old. The same lag applies to the reporting of cancer statistics across Australia [7]. More resources might be required to reduce this time lag, and provisional data could be made available with the necessary caveats. More broadly, it has been suggested that there is a need to streamline processes for approvals and access to administrative health datasets. These time lags are one of the inherent challenges of using administrative data for health services research [8].

## Limitations

The primary purpose of the non-registry data sources used in this study are administrative and not specifically for cancer identification or recording, so they should be used for this purpose with caution. Furthermore, these data sources don’t include disease stage or the actual date of diagnosis, which are important for studies assessing survival or the appropriateness or timeliness of treatment. The 45 and Up Study had a participation rate of ~ 18% and is not directly representative of the general population [2], so while the results are representative of these cases during the study period, they might not be representative of all cases or those diagnosed in later time periods. Another limitation is that to be identified in the hospital data, a person must have had at least one hospitalisation at or after diagnosis. Therefore the “missed” cases might be more commonly people with less health system contact, such as those with unknown disease stage or living in more remote areas. This means that using hospital data could attenuate estimates of relative risk in analyses of cancer-related exposures, due to potential misclassification. Also, for lung cancer in particular, the hospital cancer diagnosis dates tended to lag behind the actual diagnosis date, which may impact time-related analyses. Furthermore, the sensitivity and PPV estimates may be less accurate for people diagnosed at the start/end of the study period due to restricted follow-up time [1]. Finally, the applicability to other settings, particularly internationally, will depend on the information recorded in hospital databases and local data conventions.

## Data Availability

The data cannot be made available here as they are third party data not owned or collected by the authors and on-provision is not permitted, as it would compromise the patients’ confidentiality and participants’ privacy. However the data are available from the relevant data custodians for approved research projects—data access enquiries can be made to the Sax Institute (see https://www.saxinstitute.org.au/our-work/45-up-study/governance/ for details). Other researchers would be able to access these data in the same manner as the authors. The authors did not have any special access privileges that others would not have.

## Abbreviations

APDC: Admitted Patient Data Collection
COD-URF: Cause of Death Unit Record File
ICD10: International Classification of Diseases 10th Edition
NSW: New South Wales
NSWCR: New South Wales Cancer Registry
PPV: positive predictive value

## References

[1] Goldsbury D, Weber M, Yap S (2017). Identifying incident colorectal and lung cancer cases in health service utilisation databases in Australia: a validation study. BMC Med Inform Decis Mak.

[2] Banks E, Redman S, Jorm L (2008). Cohort profile: the 45 and up study. Int J Epidemiol.

[3] Centre for Health Record Linkage (website). http://www.cherel.org.au. Accessed 17 Jul 2019.

[4] Department of Health and Aged Care. Measuring remoteness: accessibility/remoteness index of Australia (ARIA). Revised Ed. Occasional Paper New Series No. 14. 2001.

[5] Howlader N, Ries LAG, Mariotto AB (2010). Improved estimates of cancer-specific survival rates from population-based data. JNCI.

[6] Weber M, Yap S, Goldsbury D (2017). Identifying high risk individuals for targeted lung cancer screening: independent validation of the PLCOm2012 risk prediction tool. Int J Cancer.

[7] Australian Institute of Health and Welfare 2019. Cancer in Australia 2019. Cancer series no. 119. Canberra, Australia.

[8] Langton JM, Blanch B, Drew AK (2014). Retrospective studies of end-of-life resource utilization and costs in cancer care using health administrative data: a systematic review. Palliat Med.

